# Preparing for the next viral threat with broad-spectrum antivirals

**DOI:** 10.1172/JCI170236

**Published:** 2023-06-01

**Authors:** Marwah Karim, Chieh-Wen Lo, Shirit Einav

**Affiliations:** 1 Division of Infectious Diseases and Geographic Medicine, Department of Medicine, and; 2 Department of Microbiology and Immunology, Stanford University School of Medicine, Stanford, California, USA.; 3 Chan Zuckerberg Biohub San Francisco, San Francisco, California, USA.

## Abstract

There is a large global unmet need for the development of countermeasures to combat hundreds of viruses known to cause human disease and for the establishment of a therapeutic portfolio for future pandemic preparedness. Most approved antiviral therapeutics target proteins encoded by a single virus, providing a narrow spectrum of coverage. This, combined with the slow pace and high cost of drug development, limits the scalability of this direct-acting antiviral (DAA) approach. Here, we summarize progress and challenges in the development of broad-spectrum antivirals that target either viral elements (proteins, genome structures, and lipid envelopes) or cellular proviral factors co-opted by multiple viruses via newly discovered compounds or repurposing of approved drugs. These strategies offer new means for developing therapeutics against both existing and emerging viral threats that complement DAAs.

## Introduction

Over 200 viruses are known to cause disease in humans, yet currently approved antiviral drugs are available to treat only about 10 of these viral infections. The past decade has underscored the global threat posed by emerging viruses. Spillovers from animals to humans have resulted in several Ebola virus disease (EVD) outbreaks, the Middle East respiratory syndrome (MERS) outbreak, and possibly the current coronavirus disease 2019 (COVID-19) pandemic. Global warming, increased urbanization, and air travel have contributed to the spread of vector-borne viruses endemic to various parts of the world, including dengue virus (DENV), estimated to infect 400 million people in over 128 countries, and Zika virus (ZIKV), the causative agent of a 2015 outbreak. Moreover, political instability in various parts of the world continues to pose risks to our military forces and civilians from potential spread of biothreat agents, such as poxviruses — whose natural spread caused the ongoing monkeypox virus (MPXV) outbreak — and Venezuelan equine encephalitis virus (VEEV) (1, 2). There is thus a huge unmet need for the development of effective therapeutics for the treatment of existing and newly emerging viral infections.

**Figure 1:**
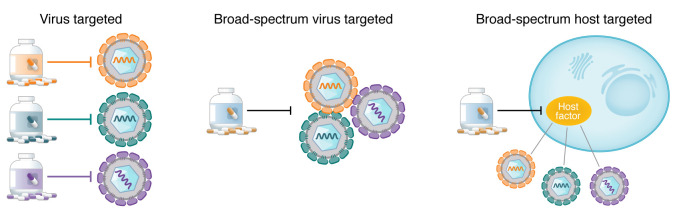
Toward broad-spectrum antivirals. Antiviral drugs that selectively inhibit unique viral proteins typically provide a narrow-spectrum solution (left), whereas broad-spectrum drugs can restrict multiple viruses by inhibiting either common viral functions or structures (middle) or host factors commonly required by several viruses (right). Adapted with permission from *Science* (204).

Most approved antivirals target viral enzymes, particularly proteases and polymerases (Figure 1). Such direct-acting antivirals (DAAs) have shown tremendous utility for the treatment of hepatitis C virus (HCV) and human immunodeficiency virus type 1 (HIV-1) infections, and more recently COVID-19. However, this approach to drug development has several major limitations. First, the spectrum of coverage provided is typically narrow, ranging from a single viral genotype to a few related viruses at best. Moreover, this approach is not scalable to address the large unmet need. It takes, on average, an 8- to 12-year timeline (3) and an average cost of over $2 billion to develop a new drug. Thus, targeting viruses individually is expensive and slow. While timely, effective efforts were noted during the COVID-19 outbreak, the rapid rollout of nirmatrelvir, for example, was enabled by accelerated derivatization of an existing series of SARS-CoV-1 main protease (Mpro) inhibitors. No such DAAs are, however, currently available for the majority of viral families. The inability to predict the next emerging viral infection is another limitation, hampering adequate global health protection and national security preparedness. Lastly, when used individually, treatment with conventional DAAs often results in rapid emergence of drug resistance, complicating monotherapy regimens for HIV, HCV, and influenza A virus (IAV). In the case of SARS-CoV-2, escape mutations conferring high-level resistance to remdesivir and nirmatrelvir have already been selected in vitro and identified in circulating strains (4, 5). While combining drugs that target distinct viral functions can overcome viral resistance, as exemplified by HIV and HCV treatment, developing such “cocktail” regimens for multiple acute infections is not feasible.

An alternative solution is the development of broad-spectrum antiviral drugs. One advantage of this approach is reduced time and cost associated with the early stages of drug development per approved indication. It can also diminish the clinical risks in more advanced stages of development. The off-label use of approved antivirals against new viral indications can provide further economic incentives, as these drugs were already rigorously tested for toxicity, pharmacokinetics, pharmacodynamics, dosing, etc. These advantages have been recently demonstrated by the repurposing of remdesivir and molnupiravir — originally developed to treat EVD and VEEV, respectively — for the treatment of COVID-19 (6, 7). Importantly, this approach can facilitate readiness for future outbreaks of newly emerging pathogens. Broad-spectrum antivirals could also be used to treat rare viral infections for which no drug is available. Lastly, a broad-spectrum antiviral could be administered before a viral threat has been accurately diagnosed, increasing the likelihood of viral control, with implications for front-line health care providers and military personnel.

Broad-spectrum antiviral activity can be achieved by targeting of viral components or cellular factors required for the replication of multiple viruses (Figure 1). The latter approach could complement DAAs, such as by conferring synergistic antiviral effects, as recently demonstrated by a combination of molnupiravir (DAA) with camostat mesylate (host-targeted) (8). Here, we summarize recent efforts to characterize the therapeutic potential and biological rationale of representative approaches under these categories. Notably, we define broad-spectrum coverage as activity against viruses from at least two unrelated viral families.

## Broad-spectrum DAAs

**Figure 2:**
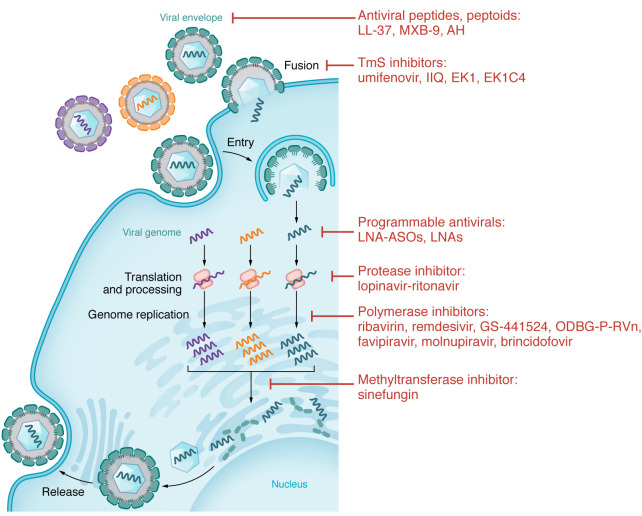
Approved and experimental direct-acting compounds with broad-spectrum antiviral activity. Depicted here is a generic viral life cycle. Examples of classes of inhibitors with broad-spectrum antiviral activity are connected to the specific stages of the viral life cycle or cellular process they target.

Most virally encoded proteins show extensive sequence and structural diversity. Thus, the spectrum of coverage typically provided by DAAs is narrow, ranging from several serotypes or variants of the same virus to a few related viruses at most, as exemplified by paritaprevir and Paxlovid — HCV and SARS-CoV-2 inhibitors, respectively. Accordingly, the number of DAA classes showing promise in preclinical and clinical studies has been limited to date (Figure 2 and Table 1).

### Targeting viral polymerases.

The structure of the catalytic units of most RNA-dependent RNA polymerases is highly conserved across viral families, making them attractive targets for broad-spectrum antivirals (9). Discovered in the 1970s, the nucleoside analog ribavirin introduced the concept of broad-spectrum antivirals. Several mechanisms of ribavirin’s antiviral action have been demonstrated, including inhibition of viral RNA or DNA synthesis (10). Ribavirin was shown to suppress the replication of multiple viruses in vitro and to confer protection from multiple emerging viral pathogens, including filo- and arenaviruses, in nonhuman primates (NHPs) (11, 12). Ribavirin is approved for the treatment of HCV infection in combination drug regimens (13) and of respiratory syncytial virus (RSV) infection in immunocompromised patients (14). Moreover, ribavirin reduced mortality when tested in over 1,800 patients infected with Lassa virus, albeit the comparative arm was historic controls (15). Ribavirin treatment, however, did not impact COVID-19 outcomes (16), and its clinical utility for other viral infections remains to be determined.

In the past decade, several chemically distinct, next-generation nucleotide and nucleoside analogs have demonstrated broad-spectrum antiviral activity (reviewed in ref. 17). One example is remdesivir, an intravenously administered nucleotide analog prodrug that suppresses viral RNA replication via delayed chain termination (18). Remdesivir was initially developed for treatment of EVD after demonstrating effective suppression of viral replication in human primary cells and 100% protection from lethality in NHPs (6). Contrastingly, however, in a randomized multi-intervention trial (the PALM study) in 681 EVD patients, remdesivir treatment did not reduce viremia and in fact increased mortality rate relative to monoclonal antibodies (19). Remdesivir has shown activity against other hemorrhagic viruses, including Nipah virus, albeit thus far in preclinical models only (20). Remdesivir has also shown utility for the treatment of respiratory viruses, suppressing replication and/or tissue injury in NHP models of RSV, and coronaviruses (21–23). Remdesivir was therefore one of the first repurposed agents to be tested clinically for COVID-19 treatment. Following inconclusive studies (24, 25), in a phase III trial (Adaptive Covid-19 Treatment Trial [ACTT-1]) involving 1,062 hospitalized patients with SARS-CoV-2 pneumonia, remdesivir shortened the median recovery time and reduced mortality rate relative to placebo without causing severe side effects (26). Based on these findings and its prior de-risking in human trials, remdesivir was the first drug to receive FDA approval for COVID-19 treatment. Nevertheless, the need to deliver remdesivir intravenously has somewhat limited its global application, prompting the design of analogs for oral delivery (27). VV116, one such analog, potently suppresses SARS-CoV-2 replication and improves oral bioavailability (28). In a phase III trial, VV116 demonstrated comparable time to clinical recovery to Paxlovid and a favorable safety profile (29). Other oral analogs of remdesivir, such as GS-441524 (30), are undergoing development.

Favipiravir (T-705) is a nucleoside analog whose active form gets incorporated into the nascent viral RNA strand, inducing lethal mutagenesis (31, 32). In cell culture models, favipiravir has demonstrated moderate antiviral activity against IAV and VEEV, and weak activity against SARS-CoV-2 and Ebola virus (EBOV) (EC_50_ values over 60 μM) (33–35). While high concentrations are required to achieve therapeutic levels in humans, by inhibiting its own metabolism, favipiravir increases its cellular uptake (reviewed in ref. 36). Favipiravir was approved for flu treatment in Japan in 2014 and for the treatment of COVID-19 in China and India after demonstrating some benefits in early studies (37–39). However, in prospective randomized COVID-19 studies, favipiravir showed no clinical benefit over placebo (40, 41). Beyond respiratory viral infections, favipiravir protected EBOV-infected mice from lethality (33). Nevertheless, while it reduced viral load and prolonged survival in a retrospective EBOV study, it showed no benefit in a phase II trial (42, 43). Conversely, favipiravir increased viral clearance and reduced mortality rate in a trial involving 145 patients infected with a different hemorrhagic virus: the phlebovirus severe fever with thrombocytopenia syndrome virus (SFTSV) (44). The mutagenesis pattern of SFTSV in serum samples was comparable to that observed in preclinical models, confirming favipiravir’s mechanism of action (44).

Molnupiravir is another orally bioavailable nucleoside analog whose incorporation into the viral genome causes lethal mutagenesis (45). Designed to inhibit VEEV (7), molnupiravir is rapidly distributed to brain tissue and protects mice from a lethal VEEV challenge (46). Molnupiravir demonstrated activity in animal models of EBOV and respiratory viral infections, including IAV and pandemic coronaviruses (47–50). Yet, whereas in earlier phase II and III trials in mild-to-moderate COVID-19 patients, molnupiravir accelerated SARS-CoV-2 clearance and reduced mortality (51, 52), prompting its Emergency Use Authorization as a second-line COVID-19 treatment, in a more recent phase II trial, molnupiravir’s antiviral effect was inconclusive (53).

DNA-dependent DNA polymerases have also been shown to be amenable to broad-spectrum inhibition. Brincidofovir is an oral nucleoside analog, prodrug of cidofovir, whose incorporation into the elongating viral DNA by the viral polymerase interrupts DNA replication via chain termination and/or direct inhibition (54). Brincidofovir has demonstrated in vitro and in vivo activity against multiple DNA viruses (55). Based on efficacy data in animal models, brincidofovir was approved for the treatment of smallpox in 2021 (56). Nevertheless, brincidofovir showed no virologic benefit in patients infected with MPXV in a retrospective observational study, and treatment was complicated by liver toxicity (57). In phase II and III trials in allogeneic hematopoietic cell transplant recipients, brincidofovir reduced adenovirus viremia and prevented cytomegalovirus (CMV) viremia (58–60). Yet a trend toward reduced mortality was observed in adenovirus viremic patients only, and treatment was complicated by acute graft-versus-host disease (58, 60). Independent of polymerase inhibition, suppression of EBOV replication in vitro by brincidofovir is thought to be mediated by its lipid side chain (61), yet its clinical utility for this indication remains to be determined, as it has been studied only anecdotally to date (62, 63).

These and other examples highlight the broad-spectrum potential of polymerase inhibitors.

### Targeting other viral enzymes.

While the unique substrate preference of viral (versus cellular) proteases can facilitate relatively selective inhibition, their large diversity across viral families has limited their potential as targets for broad-spectrum antivirals. Approved for the treatment of HIV-1 infection, lopinavir-ritonavir combination (Kaletra) was shown to bind the substrate-binding pocket of SARS-CoV-1’s main protease (Mpro) (64) and suppress SARS-CoV-2 replication in vitro (65) — somewhat surprising findings since coronaviruses encode cysteine proteases whereas HIV-1 encodes an aspartic protease. However, while potential benefit in reducing lung injury was demonstrated in a retrospective study in SARS-CoV-1–infected patients treated with a combination of lopinavir-ritonavir and ribavirin (66), no such benefit was observed in SARS-CoV-2–infected ferrets and humans (67–69). Thus, the overall broad-spectrum utility of viral protease inhibitors to date has been limited.

Targeting of viral methyltransferases (MTases) — enzymes essential for capping the mRNA 5′ ends of some viruses for efficient translation and evasion of immune responses — has also been explored (70). Competition with *S*-adenosyl-l-methionine (SAM) on MTase binding, such as by sinefungin, was shown to suppress MTases of alphaviruses, flaviviruses, and SARS-CoV-2 in vitro (71–73), yet severe toxicity in preclinical models, attributed to lack of selectivity, hampered the clinical development of this approach (74). Greater selectivity achieved by targeting of conserved pockets near the SAM-binding site, combined with conservation of MTase structure within viral families, has enabled the discovery of investigational pan-flaviviral inhibitors with reduced toxicity, yet the feasibility of developing MTase inhibitors with activity across viral families is low (75–78). Similarly, the broad-spectrum potential of inhibitors targeting other viral enzymes including exonucleases and helicases remains to be defined.

### Targeting viral fusion proteins, lipid envelope, and genome.

Targeting class I fusion glycoproteins of enveloped viruses is another strategy explored for its broad-spectrum potential. The transmembrane subunit (TmS) of these proteins is highly conserved and thus an attractive target for broad viral inhibition (reviewed in ref. 79). Umifenovir (Arbidol), one example of such a strategy, binds to a hydrophobic pocket in the stem region of the TmS of IAV hemagglutinin, thereby blocking viral fusion with endosomal membranes (80). Umifenovir has shown efficacy in cell culture and animal models of IAV infection (81), and in a phase IV trial in 359 flu patients (82), leading to its approval for flu treatment in Russia and China. Umifenovir suppresses replication of other RNA viruses in vitro, albeit with moderate EC_50_ values (5.7–32.3 μM) (81). Whereas an open-label study suggested potential benefit of umifenovir treatment in 100 COVID-19 patients (83), a retrospective study showed increased mortality in severe COVID-19 patients (84), and the results of a phase IV randomized study are unavailable (ClinicalTrials.gov NCT04260594), making it difficult to draw conclusions. Beyond small molecules, suppression of viral fusion by α-helical lipopeptides that disrupt α-helix–mediated interactions of the TmS is another strategy that shows broad-spectrum potential. IIQ, one such candidate, suppresses the replication of multiple RNA viruses in vitro and achieves good exposure levels in rats (85). EK1 and EK1C4, peptides that target the heptad repeat-1 (HR1) domain of TmS of human coronaviruses, have shown prophylactic and therapeutic effects when administered intranasally to mice infected with coronaviruses (86). However, the broad-spectrum potential of these and other fusion-suppressing peptides demonstrating activity against specific viruses (87, 88) remains to be defined.

The viral envelope is another emerging target for broad-spectrum antiviral interventions. The utilization of antimicrobial peptides has been challenged by cytotoxicity resulting from a lack of selectivity to the viral lipid envelope and by rapid degradation by cellular proteases. Nevertheless, recent efforts indicate that harnessing differences between the membrane curvature of viral particles and that of cells can achieve selectivity, and that modifying peptides — such as by stapling or designing synthetic peptidomimetics that resist proteolytic degradation (peptoids) — can improve biostability. Indeed, various amphipathic, α-helical (AH) peptides and self-assembling peptoids have demonstrated effective viral membrane lysis and abrogation of infectivity without impacting cellular viability (89, 90). In a mouse model, an AH peptide suppressed ZIKV infection and reduced inflammation and blood-brain barrier injury (91). LL-37 and MXB-9, with activity against multiple viruses in cultured cells and/or mice infected with SARS-CoV-2 pseudovirus (90, 92), provide additional proof of concept for the potential utility of this approach.

Targeting of the viral genome as a broad-spectrum antiviral approach has also shown promise recently. “Programmable antivirals,” such as locked nucleic acids (LNAs) and LNA antisense oligonucleotides (LNA ASOs) targeting highly conserved viral RNA structures involved in viral packaging or replication, are one example of this approach. Such LNAs and LNA ASOs suppressed replication of HCV, IAV, and SARS-CoV-2 in vitro (93–95), and reduced mortality, viral load, and/or transmission (94, 95) in mice infected with SARS-CoV-2 and IAV (94).

Taken together, while the design of DAAs with activity across viral families is overall challenged by the extensive sequence and structural diversity of virally encoded proteins, targeting of viral polymerases and non-enzymatic viral functions holds promise.

## Host-targeted broad-spectrum antiviral approaches

The cellular machineries co-opted to support the life cycle of viruses are often conserved across viral families, representing attractive targets for broad-spectrum antiviral strategies. With approximately 20,000 proteins, the human proteome offers a much larger repertoire of candidate targets than a viral proteome. Indeed, the discovery of such proviral factors required by multiple viruses has been the subject of fruitful research. Aided by breakthroughs in multi-omics approaches, these efforts have led to the discovery of numerous druggable proviral factors. Some examples are discussed below.

Beyond a larger target repertoire, an important advantage of the host-targeted approach is its higher barrier to viral resistance. Since cellular targets are not under genetic control of a virus, the likelihood that escape mutations will emerge is lower than with DAAs. This advantage was demonstrated in cell culture models, such as with inhibitors targeting various cellular kinases (96–98), and in animal models, such as DENV-infected mice treated with α-glucosidase inhibitors (99). In patients, cyclophilin inhibitors and other host-targeted approaches have demonstrated longer time to resistance and lower levels of resistance than DAAs (100).

Targeting cellular functions can also provide opportunities not only to suppress viral replication but also to moderate deleterious host responses, which play key roles in the pathogenesis of multiple viral infections, including dengue, EVD, and COVID-19. Targeting p38 MAPK or ErbBs, for example, as we and others have demonstrated in preclinical models, can reduce inflammation and protect from tissue injury beyond suppression of viral replication (98, 101). Another example is enhancement of type I interferon responses contributing to the protective effect of tamoxifen treatment in vesicular stomatitis virus–infected (VSV-infected) mice (102). Lastly, since most approved drugs target cellular functions, there is an opportunity to repurpose existing drugs for antiviral indications, as was extensively explored during the COVID-19 outbreak (reviewed in ref. 103).

**Figure 3:**
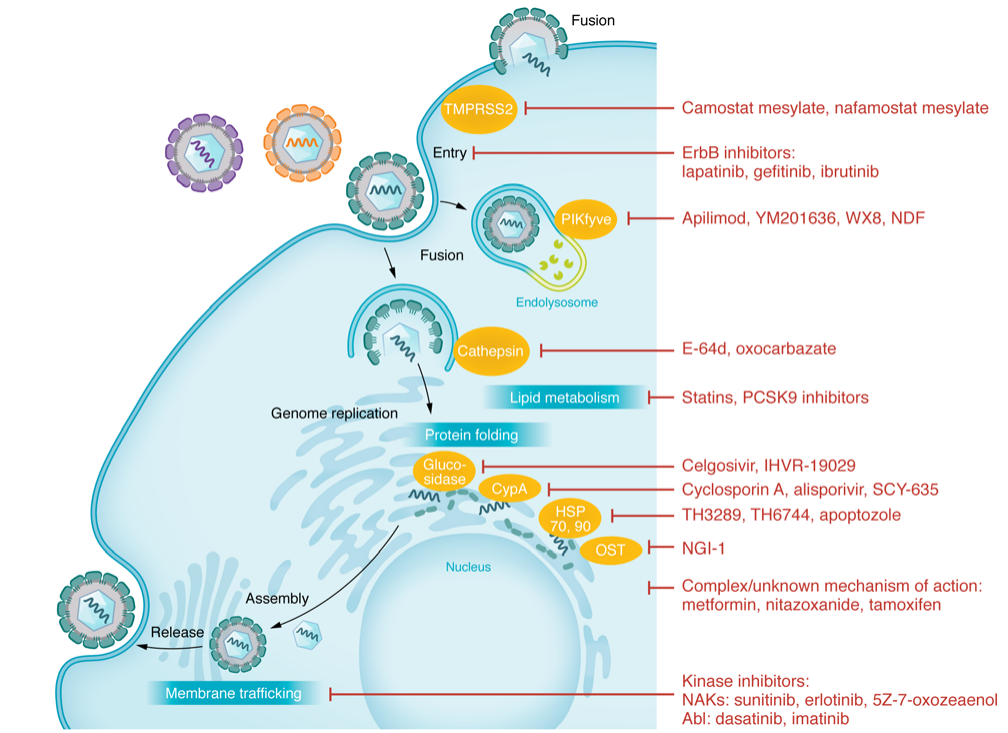
Approved and experimental host-targeted compounds with broad-spectrum antiviral activity. Depicted here is a generic viral life cycle. Examples of classes of inhibitors with broad-spectrum antiviral activity are connected to the specific stage(s) of the viral life cycle or cellular process they target.

Below are examples of classes of host-targeted approaches that show some promise (Figure 3 and Table 2).

### Targeting protein folding and transport.

Cyclosporin A (CsA) and experimental non-immunosuppressive inhibitors of cyclophilin A (CypA) — a cellular factor involved in protein folding — such as alisporivir (Debio-025) and SCY-635, suppress the replication of multiple viruses in vitro (104). Blockage of interactions between CypA and the HIV-1 nucleocapsid and HCV NS5A proteins is thought to mediate the antiviral effect (105, 106). Other mechanisms of antiviral action were reported, including suppression of HBV binding to its entry receptor (107), of coronaviral RNA synthesis (104), and of nuclear import of IAV genome (108). The effect of these compounds in mouse models has been variable (100), yet prevention of disease progression was demonstrated in mice infected with coronaviruses (109). Accordingly, transplant recipients receiving CsA treatment for their underlying condition experienced reduced morbidity and mortality upon SARS-CoV-2 infection (110). Whereas alisporivir significantly reduced viremia in chronically infected HCV patients, a phase III trial was terminated due to toxicity.

α-Glucosidase is another protein required for proper folding of proteins — including viral glycoproteins — that serves as a broad-spectrum antiviral target. Celgosivir and other iminosugars are competitive substrates for α-glucosidases with activity against multiple viruses in cultured cells (111). These inhibitors have demonstrated efficacy in murine models of RNA and DNA viruses (111, 112). The utility of celgosivir for the treatment of dengue infection is currently being explored, although safety but little or no efficacy have been documented to date in a dengue pilot study and in patients infected with HCV or HIV-1 (113–115).

The molecular chaperones heat shock protein 70 (HSP70) and HSP90, involved in protein folding and transport, are also broadly required factors shown to function at temporally distinct stages of viral life cycles (116, 117). Stabilization and transport of viral proteins were among the proposed underlying mechanisms (117, 118). Pharmacological inhibition of HSP70 by TH3289 blocked replication of flaviviruses, coronaviruses, and Crimean-Congo hemorrhagic fever virus in vitro (116, 119). In murine models of ZIKV and Chikungunya virus (CHIKV) infections, small-molecule inhibitors of these chaperones reduced viral titers, inflammation, and/or mortality (120, 121). While thus far demonstrated with tool compounds only, these examples provide evidence for the potential utility of targeting HSPs.

Oligosaccharyltransferase (OST), an endoplasmic reticulum protein complex that catalyzes N-glycosylation, was discovered as a candidate antiviral target via CRISPR screens for flaviviral proviral factors (122). OST subunits interact with DENV nonstructural proteins and are required for viral RNA replication (122). NGI-1, a small-molecule inhibitor of OST, has shown antiviral activity against flaviviruses and more recently HSV-1 and SARS-CoV-2 (123–125). Interestingly, whereas the anti-DENV activity is independent of the canonical role of OST in N-linked glycosylation, the anti-IAV effect is associated with reduced hemagglutinin (HA) and neuraminidase (NA) glycosylation (123). A concern was recently raised that glycome-modified viruses generated upon NGI-1 treatment can reduce antibody responses in IAV-infected mice and requires further investigation (126).

### Targeting cellular kinases.

Multiple cellular kinases are hijacked by viruses, representing candidate targets for broad-spectrum antivirals (127). The epidermal growth factor receptor family of tyrosine kinases (ErbB1, 2, 4) is one example. A requirement for ErbBs was documented in the entry and/or post-entry stages of multiple viruses (128). Several anticancer ErbB inhibitors, including gefitinib, demonstrate activity against HCV, human cytomegalovirus (HCMV), poxvirus, and Lassa virus in cultured cells (129–133), and CMV in guinea pigs (132). In human lung and brain organoid models of SARS-CoV-2 and VEEV infections, respectively, we have recently shown that, beyond suppressing viral replication, lapatinib, an anticancer pan-ErbB inhibitor, protects from virus-induced activation of pathways implicated in non-infectious tissue injury downstream of ErbBs, proinflammatory cytokine production, and epithelial or blood-brain barrier injury (98). Moreover, we have validated ErbB inhibition as the mechanism of antiviral action (98). Remarkably, ibrutinib, a BTK inhibitor with potent pan-ErbB activity (134), has demonstrated protection from progression to severe COVID-19, albeit in a small number of patients (135), highlighting that clinical evaluation of these ErbB inhibitors is warranted.

The numb-associated (serine/threonine) kinases (NAKs) — AAK1, BIKE, GAK, and STK16 — have also been studied as targets for broad-spectrum antivirals. We have demonstrated a requirement for NAKs in the regulation of intracellular cotrafficking of specific cellular cargo adaptor proteins with viral particles during entry, assembly, and/or release of HCV, DENV, EBOV, and SARS-CoV-2 (96, 97, 136, 137). Approved anticancer drugs with potent anti-NAK activity, including sunitinib-erlotinib combinations, 5Z-7-oxozeaenol, and chemically distinct more selective inhibitors, demonstrate broad-spectrum antiviral activity against eight viral families in vitro (96, 97, 137–139). A combination treatment with sunitinib-erlotinib was shown to protect mice from DENV and EBOV challenges (96, 138). Inhibition of intracellular membrane trafficking regulated by NAKs was validated as an important mechanism of antiviral action (96, 97, 140). The safety and efficacy of NAK inhibition for the treatment of viral infections in humans remain to be determined.

Lipid kinases have also been shown to be required for effective replication of multiple viruses. For example, the endosomal phosphatidylinositol-3-phosphate 5-kinase (PIKfyve) (141) has been implicated in the entry of filoviruses, Lassa virus, and coronaviruses (142). The PIKfyve inhibitors apilimod and YM201636 suppress trafficking and maturation of endolysosomes, preventing viral fusion and/or egress (142, 143). Apilimod is currently being studied as a COVID-19 therapeutic (NCT04446377). Whereas a suboptimal pharmacokinetic profile (144, 145) limits its development, the excellent safety profile demonstrated with apilimod in clinical trials for inflammatory diseases has de-risked PIKfyve as a target (146, 147). While two chemically distinct small molecules with anti-PIKfyve activity were recently shown to increase SARS-CoV-2–induced pathology in a mouse model, since their selectivity has not been reported, it is possible that other targets have mediated this effect (148). Further evaluation of the potential of PIKfyve inhibition in other animal models and ideally human organoid models is therefore warranted. Pharmacological inhibition of other lipid and protein kinases by approved and investigational compounds has also shown promise in vitro with variable results in animal models (reviewed in ref. 149).

### Targeting cellular proteases.

Proteases are another group of cellular enzymes co-opted by viruses. Influenza viruses and coronaviruses, for example, rely on proteases, such as TMPRSS2 and cathepsins, for cleavage and activation of their surface glycoproteins (150, 151). Among cellular protease inhibitors showing antiviral activity, camostat mesylate and nafamostat mesylate, oral serine protease inhibitors approved for the treatment of chronic pancreatitis and other conditions (152), have shown TMPRSS2-dependent suppression of viral fusion in vitro (151) and protection in mouse models of IAV and coronaviral infections (153, 154). However, when studied for the treatment of COVID-19 patients, these compounds had no significant impact on clinical outcomes (155, 156). Thus, the evaluation of other strategies targeting cellular proteases for the treatment of viral infections is warranted.

### Targeting lipid metabolism.

Cholesterol-lowering drugs, like statins, have demonstrated in vitro activity against HCV, attributed to their effect on lipid biosynthesis. Indeed, antiviral activity in cells was reversed upon addition of mevalonate or geranylgeraniol, and resistance to these drugs coincided with an increase in HMG-CoA reductase level — statins’ target (157). Nevertheless, a variable, modest, and short-lived effect was demonstrated in HCV patients when statins were combined with peginterferon-ribavirin (158). Beyond HCV, statins have demonstrated efficacy in animal models of multiple viral infections, including respiratory viruses, CMV, HIV-1, and DENV (159, 160). Owing to their ability to restore endothelial stability, statins were used, albeit in a non-formal study, in combination with an angiotensin receptor blocker for treating EVD, an infection whose pathogenesis is associated with endothelial dysfunction — showing reduced mortality in 100 patients (161). Recently, reduced morbidity and mortality were documented also in COVID-19 patients with statin prescriptions, albeit in observational studies only (162). Inhibitors of proprotein convertase subtilisin kexin type 9 (PCSK9), such as the monoclonal antibodies alirocumab and evolocumab, represent another class of lipid-lowering agents shown to suppress DENV replication in vitro and reduce mortality and inflammation in severe COVID-19 patients (163, 164). Whereas statins showed no antiviral activity in dengue patients (165), PCSK9 inhibitors may offer greater protection given the recent discovery that PCSK9 expression is induced by DENV infection in cells residing in physiologically hypoxic conditions and is increased in severe dengue patients, reducing cholesterol uptake and dampening susceptibility to statins (163).

### Host-targeted approaches with complex mechanisms of action.

Metformin, an approved oral drug for the treatment of diabetes, has demonstrated potent antiviral activity against multiple viruses in vitro. Activation of AMP-activated protein kinase–dependent (AMPK-dependent) type I interferon signaling was proposed as an underlying mechanism in DENV and HCV infections (166, 167). Metformin reduced morbidity and mortality in mice infected with DENV and IAV, but not ZIKV (168, 169). Diabetic patients on metformin treatment were found to have lower morbidity and mortality upon influenza virus infection (170) and a trend toward reduced mortality when infected with SARS-CoV-2 (171). Contrastingly, metformin showed no clinical benefit in nondiabetic COVID-19 patients (172). The therapeutic potential of metformin in reducing HIV-1 reservoirs and combating DENV infection is currently being studied clinically (173, 174).

Tamoxifen and other inhibitors of the estrogen receptor (ER) approved for the treatment of breast cancer inhibit the replication of multiple RNA and DNA viruses in vitro (175). The proposed mechanisms of antiviral action include blockage of a chloride channel required for HSV-1 entry; endosomal/lysosomal proteins required for EBOV entry; SARS-CoV-2 spike-mediated membrane fusion (176, 177); and binding of ER to HCV and CHIKV polymerases (178, 179). In rodent models of VSV, EBOV, CHIKV, and SARS-CoV-2 infections, treatment with ER antagonists reduced viral titers, inflammation, and/or mortality (175, 179, 180). Treatment with ER antagonists in humans shortened the time of SARS-CoV-2 shedding (181), reduced HCV viremia but not the resulting liver inflammation (NCT00749138), and did not impact HIV-1 viremia (182). Thus, further studies are required to define the clinical utility of ER antagonists as antivirals.

Nitazoxanide, approved for the treatment of parasitic infections, is another candidate drug for repurposing with a complex mechanism of antiviral action. Nitazoxanide suppresses replication of multiple RNA viruses in vitro and in vivo (183, 184). While the precise target remains unknown, several mechanisms of action have been proposed, such as blocking of the maturation of the influenza hemagglutinin (185) and the coronaviral spike proteins (186, 187) and, in the case of HCV and HBV infections, blocking of protein kinase R–mediated phosphorylation of eIF2α (183, 188). Nitazoxanide modestly reduced the time to resolution of flu symptoms in a phase II trial and is currently being evaluated in a phase III trial for this indication (189). Whereas the addition of nitazoxanide to peginterferon-ribavirin improved sustained virologic responses in HCV patients in a phase II trial (190), no such improvement was observed in a phase III trial in genotype 4–infected patients (191). In a recent randomized, double-blind pilot study in 50 COVID-19 patients, nitazoxanide shortened hospitalization, accelerated viral clearance, and reduced inflammatory cytokine production (192), warranting a larger-scale study.

## Ongoing challenges and future perspectives

Collectively, these examples highlight the potential held in expanding the repertoire of candidate targets from viral proteins to other viral elements and to cellular functions, and provide proof of concept for the potential utility of broad-spectrum antiviral strategies. Nevertheless, major challenges remain to be overcome to expand the clinical applications of these strategies.

Toxicity is a major concern, particularly in targeting cellular factors, requiring careful safety investigations. For example, dasatinib, an inhibitor of the Src and c-Abl kinases, has demonstrated broad-spectrum antiviral activity in cultured cells, yet in a murine model of vaccinia virus, it induced immunosuppression rather than protection (193, 194). Nevertheless, since all non-infectious human diseases are treated with drugs targeting cellular functions, the increased risk posed by host-targeted antivirals is theoretical and can be potentially mitigated by the identification of a therapeutic window within which a drug level is sufficient to suppress viral replication without causing cellular toxicity. Directing the use of host-targeted approaches toward acute viral infections requiring shorter duration of treatment should further help limit toxicity. Indeed, chronically infected HCV patients receiving alisporivir unexpectedly developed fatal cases of pancreatitis during a phase III trial, albeit after several months of treatment (195). Broad-spectrum DAAs are also not devoid of toxicity: brincidofovir administration to patients infected with MPXV was complicated by liver toxicity (57), and caution is needed with favipiravir and molnupiravir treatment due to teratogenicity (196). Significant toxicity caused by lack of selectivity to the viral targets has hampered the clinical development of some DAAs, such as sinefungin targeting cellular MTases and nucleoside analogs targeting mitochondrial RNA polymerase (74, 197).

Another challenge of host-targeted approaches is that the mechanism of antiviral action is often elusive and the molecular targets underlying the antiviral effect are unvalidated. This challenge is driven in part by the complex network of interactions in which cellular proteins function and the limited selectivity of some of their inhibitors. For example, whereas the effect of erlotinib on HCV infection was first attributed solely to its effect on its cancer target, EGFR, inhibition of GAK, another target of erlotinib, was then shown to play a role (96, 129). The mechanism of antiviral action of some drugs, such as nitazoxanide and tamoxifen, is even less clear and is often pathogen specific (176–179, 185–188).

But the greatest challenge of all antiviral approaches is the limited translatability of protective effects observed in preclinical models into clinical benefit in humans. While this limitation would be predicted to impact primarily host-targeted approaches owing to potential differences in the sequence and/or structure of proviral factors across species, this does not appear to be the case. The translation of broad-spectrum DAAs seems to be comparably impacted. For example, remdesivir showed excellent protection from EVD in NHPs, yet no benefit in EBOV-infected patients (19). The narrow window of opportunity for therapeutic interventions in the case of acute viral infections undoubtedly contributes to these low clinical translation rates.

These challenges underscore the need to consider revising the procedures currently in place to assess antivirals. Preclinically, careful consideration of differences in pharmacological properties including pharmacokinetics and tissue distribution between species may improve the success rate of clinical translation. The use of more biologically relevant human organoids and organ-on-chip models to mimic human tissue architecture may also help address this challenge. Indeed, the use of such models is now being encouraged by the FDA (198). On the clinical front, the design of clinical studies, particularly those conducted in the setting of outbreaks, could be considerably improved. The adaptive platform design — adapted from clinical studies in cancer (199) and approved by the FDA (NCT02380625) (200) — is one solution showing promise during the COVID-19 pandemic (NCT04280705) (reviewed in ref. 103). Improving patient selection in clinical trials by targeting treatment to patients more likely to develop severe outcomes may further enhance the resolution of clinical studies. Recent breakthroughs in omics approaches and machine learning algorithms enabling the discovery of clinically usable biomarkers — such as those we and others have identified to predict progression to severe dengue infection and other severe viral infections (201–203) — may aid with this effort.

Taken together, while much progress has been achieved in the field of broad-spectrum antivirals, the need to establish a therapeutic portfolio for future pandemic preparedness is far from being met. Developing and stocking host-targeted broad-spectrum antivirals as the first line of defense, and in parallel developing DAAs for representative viruses from each major viral family — efforts currently supported by US government funding — should bring us closer to achieving this goal.

## Figures and Tables

**Table 1:**
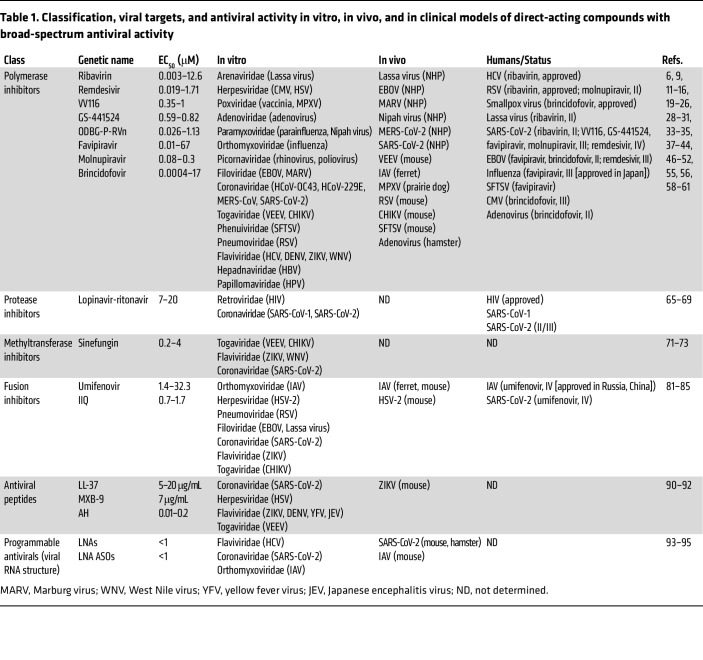
Classification, viral targets, and antiviral activity in vitro, in vivo, and in clinical models of direct-acting compounds with broad-spectrum antiviral activity

**Table 2:**
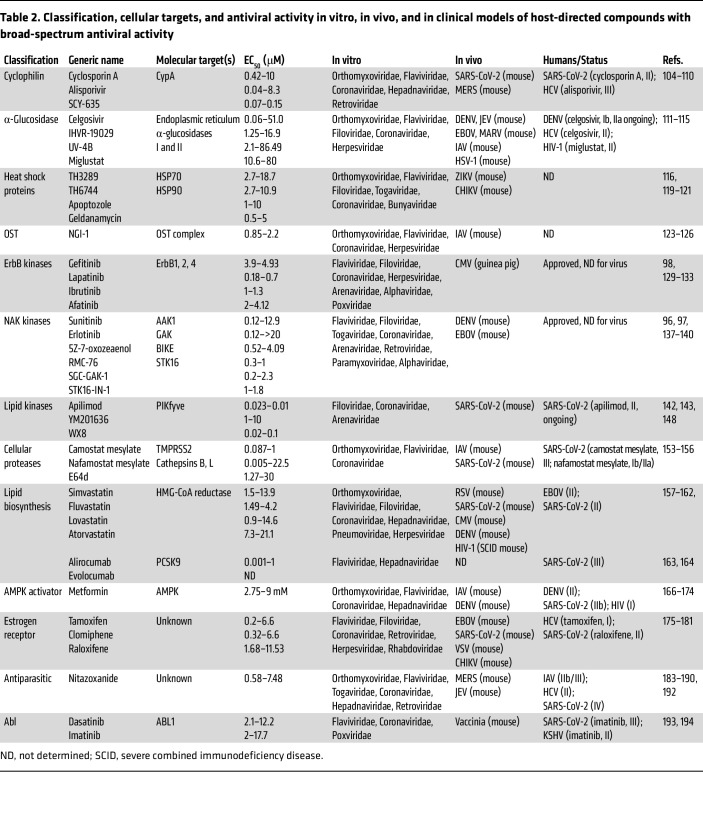
Classification, cellular targets, and antiviral activity in vitro, in vivo, and in clinical models of host-directed compounds with broad-spectrum antiviral activity

## Version 1 06/01/2023

Electronic publication
